# Supplementary material for: Reduced Zeb1 Expression in Prostate Cancer Cells Leads to an Aggressive Partial-EMT Phenotype Associated with Altered Global Methylation Patterns
Source: Int J Mol Sci. 2021 Nov 27;22(23):12840. doi: 10.3390/ijms222312840 (PMC8657557; doi:10.3390/ijms222312840)
Supplement: Supplementary file 1 [file ijms-22-12840-s001.zip › ijms-1471449-supplementary.pdf]

Supplemental Data

Reduced *Zeb1* Expression in Prostate Cancer Cells Leads to an Aggressive Partial-EMT Phenotype Associated with Altered Global Methylation Patterns

Jenna Kitz [1], Cory Lefebvre [1], Joselia Carlos [2], Lori E. Lowes [3] and Alison L. Allan [1,4,5,*]

1 London Regional Cancer Program, London Health Sciences Centre, Department of Anatomy & Cell Biology, Western University, London, ON, N6A 5W9, Canada; jkitz@uwo.ca (J.K.); clefebvre2019@meds.uwo.ca (C.L.); alison.allan@lhsc.on.ca (A.L.A.)
2 Department of Medical Biophysics, Western University, London, ON, N6A 5C1, Canada; jcarlos6@uwo.ca
3 Flow Cytometry, London Health Sciences Centre, London, ON, N6A 5W9, Canada; lori.lowes@lhsc.on.ca
4 Department of Oncology, Western University, London, ON, N6A 5W9, Canada
5 Cancer Research Laboratory Program, Lawson Health Research Institute, London, ON, N6C 2R5, Canada
* Correspondence: alison.allan@lhsc.on.ca; Tel.: +1-519-685-8600 (ext. 55134)

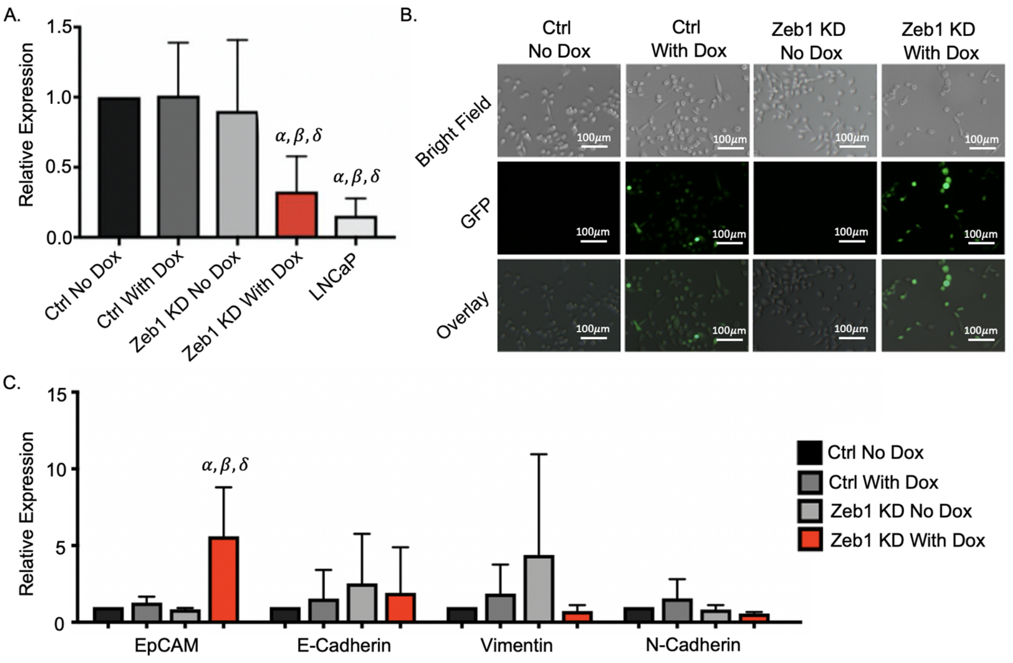

Supplemental Figure S1. *Zeb1* RNA can be inducibly knocked down in PC-3 human prostate cancer cells. Mesenchymal human PC-3 prostate cancer cells were engineered to knockdown expression of the master EMT regulator Zeb1 using the SMARTvector inducible lentiviral shRNA system (Dharmacon), which features Tet-on® induction of the target shRNA in the presence of Doxycycline (Dox). **(A)** qRT- PCR analysis of *Zeb1* mRNA expression in the presence of absence of Dox in Zeb1^{KD} or Ctrl cells, or LNCaP cells. **(B)** Immunofluorescence of Zeb1^{KD} and Ctrl cells ± Dox. Scale bars = 100 μm. **(C)** qRT- PCR analysis of *EpCAM*, *E-Cadherin*, *Vimentin*, and *N-Cadherin* mRNA expression in Zeb1^{KD} or Ctrl cells ± Dox. Data is presented as the mean ± SEM (n=3). α= significantly different than PC-3 Ctrl no Dox. β= significantly different than Ctrl with Dox. δ= significantly different than Zeb1^{KD} no Dox ($p \leq 0.05$).

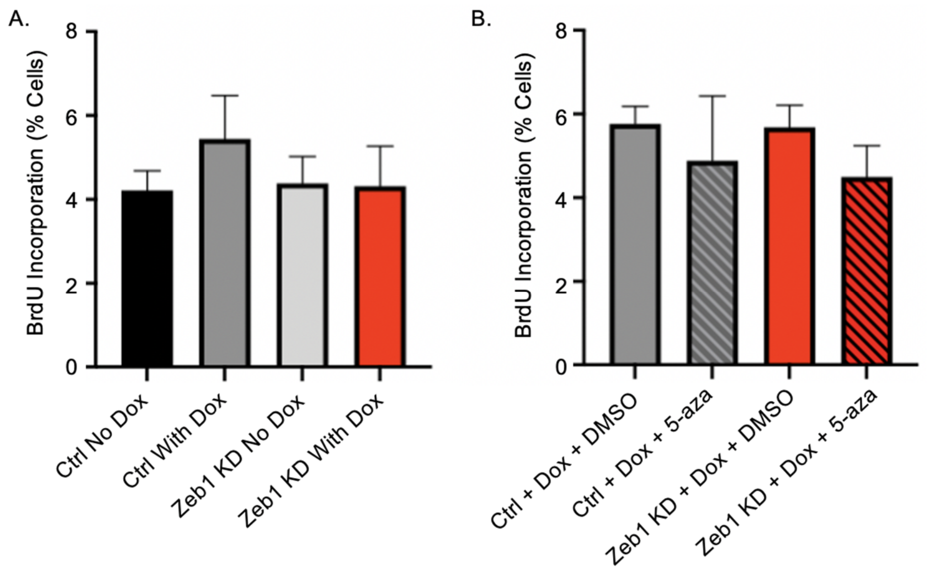

Supplemental Figure S2. Knockdown of *Zeb1* in PC-3 prostate cancer cells does not alter cell proliferation. Cells ($1.6x10^4$/well) were seeded on 8-well chamber slides with or without Dox, DMSO, and/or 5uM 5-azacytidine (5-aza), a global demethylating agent. Cells were serum starved for 3 days and then treated with media containing fetal bovine serum albumin for 24h. Cells were then treated with BrdU for 30 min and formalin fixed. Cells were incubated with a BrdU antibody overnight and visualized using 5 high-powered fields of view (FOV) using DAPI mounting media. **(A)** BrdU incorporation of Ctrl and $Zeb1^{KD}$ cells ± Dox. **(B)** BrdU incorporation of Ctrl and $Zeb1^{KD}$ cells + Dox ± DMSO or 5-aza (5uM). Data is presented as the mean ± SEM (n=3).

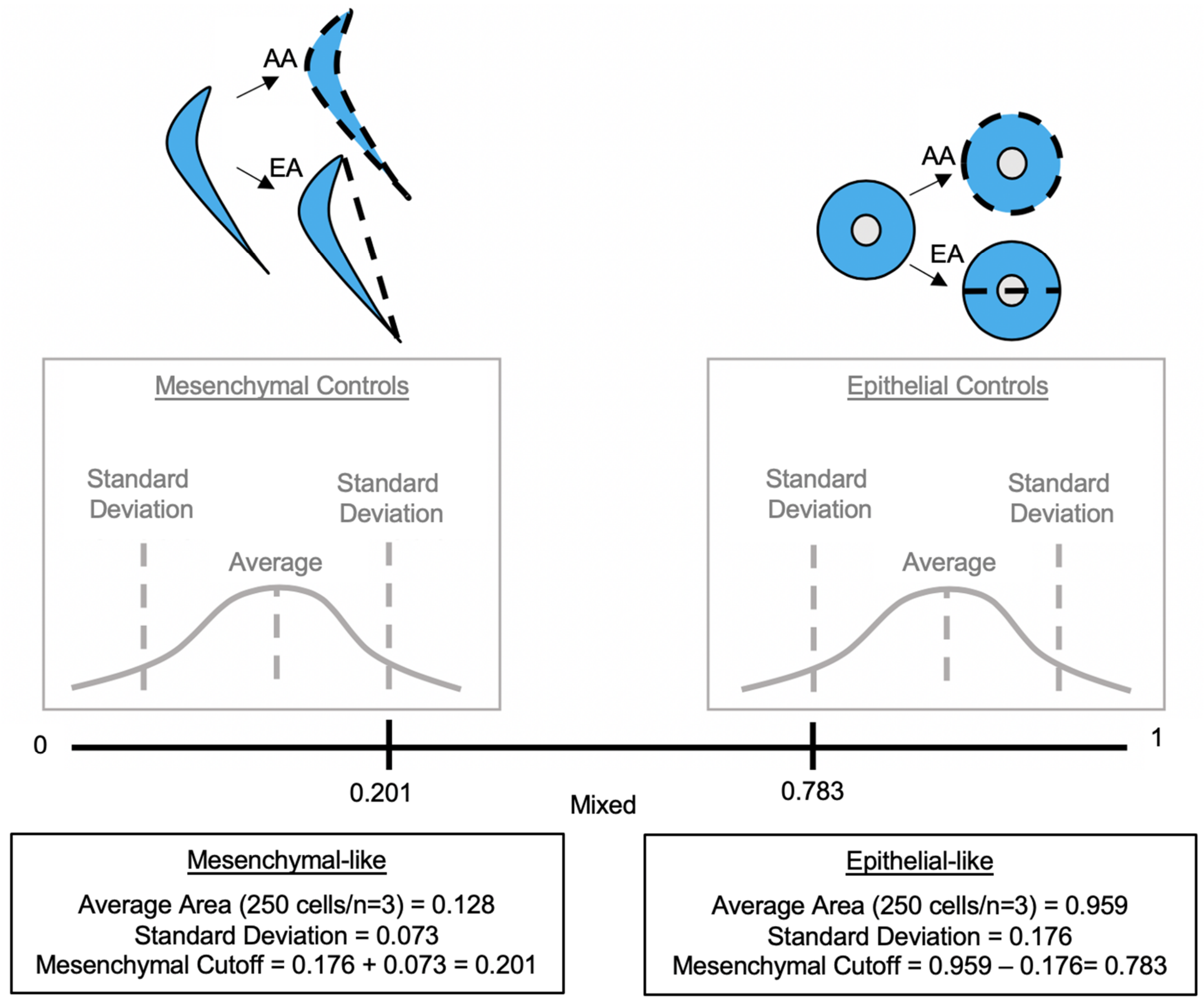

Supplemental Figure S3: Cell morphology assay calculations. Epithelial (MDA-MB-468 breast cancer cells) and mesenchymal (primary lung fibroblasts) control cells were assessed for cell shape (250 cells, n=3). The average was calculated and the "cut-off" points for a round (epithelial) cell and an elongated (mesenchymal) cell were calculated by subtracting/adding the standard deviation from/to the average. Zeb1^{KD} and Ctrl cells were then analyzed for cell shape (250 cells, n=3). Any value within the mesenchymal "cut-off" (0-0.201) was considered mesenchymal-like, any value within the epithelial "cut-off" (0.783-1) was considered epithelial-like, and any value which fell in between (0.202-0.782) was considered mixed.

Supplemental Table S1. Primary antibodies for immunoblotting.

Target Protein	1° Host	kDa	1° Conditions (Overnight @ 4℃)	2° Conditions (1 hour @ room temperature)	Reduced?	Poly/Mono Clonal
**Zeb1** (Cell Sig.- 3396)	Rabbit	200	1:500	1:1000	Yes	Mono (D80D3)
**EpCAM** (Abcam- ab32392)	Rabbit	39	1:1000	1:2000	Yes	Mono (E144)
**E-Cadherin** (BD Biosciences- 610181)	Mouse	120	1:2000	1:2000	Yes	Mono (36)
**N-Cadherin** (Abcam- ab76011)	Rabbit	100	1:1000	1:2000	Yes	Mono (EPR1791-4)
**Vimentin** (Millipore-MAB3400)	Mouse	60	1:1000	1:2000	Yes	Mono (V9)
**P-Cadherin** (Abcam- ab137729)	Rabbit	91	1:1000	1:2000	Yes	Poly
**Integrin β4** (Abcam- ab29042)	Mouse	202	1:1000	1:1000	Yes	Mono (M126)
**Actin** (Sigma- A2006)	Rabbit	42	1:5000	1:5000	Yes	Poly

Supplemental Table S2. Forward and reverse primers used for qRT-PCR

Target Gene	Forward Primer (5′ → 3′)	Reverse Primer (5′ → 3′)
Zeb1	AGCACTTGTCTTCTGTGTGATG	CAGGCTTCTCAGCTTCTGCT
EpCAM	CGACTTTTGCCGCAGCTCAGGA	GGGCCCCTTCAGGTTTTGCTCT
E-Cadherin	TGCTGATGCCCCCAATACCCCA	GTGATTTCCTGGCCCACGCCAA
N-Cadherin	TGACTCCAACGGGGACTGCACA	AGCTCAAGGACCCAGCAGTGGA
Vimentin	AACCAACGACAAAGCCCGCGTC	TTCCGGTTGGCAGCCTCAGAGA
P-Cadherin	AAGTGCTGCAGCCAAAGACAGA	AGGTAGACCCACCTCAATCATCCTC
Integrin β4	GCTTCACACCTATTTCCCTGTC	GACCCAGTCCTCGTCTTCTG
GAPDH	TCCATGGCACCGTCAAGGCTGA	GCCAGCATCGCCCCACTTGATT
